# Association of Severe Obesity, Hypertension, and Physical Activity with 24 h Heart Rate Variability in Adults

**DOI:** 10.3390/jcdd13060242

**Published:** 2026-06-02

**Authors:** Débora Andrea Castiglioni Alves, Pamela Carvalho da Rosa, Andréa Castiglioni Alves Teixeira e Silva, Joceli Fernandes Alencastro Bettini de Albuquerque Lins, Gisela Arsa, Lucieli Teresa Cambri

**Affiliations:** Postgraduate Program in Physical Education, Federal University of Mato Grosso, Cuiabá 78060-900, MT, Brazil; d.castig@terra.com.br (D.A.C.A.); gisarsa@gmail.com (G.A.)

**Keywords:** body fat, blood pressure, cardiac autonomic modulation, diabetes mellitus, dyslipidemia, heart rate, medication, parasympathetic modulation

## Abstract

**Background**: Heart rate variability (HRV), the physiological variation between consecutive heartbeats and a non-invasive marker of autonomic regulation, is associated with cardiovascular health. This retrospective cross-sectional study investigated the associations of severe obesity, hypertension, and physical activity with 24 h HRV in patients undergoing evaluation for bariatric surgery. **Methods:** A total of 1048 individuals were classified according to obesity class, hypertension diagnosis, and physical activity level. **Results**: Severe obesity was associated with lower 24 h HRV indices (*p* < 0.001) and higher odds ratio of hypertension (OR 2.04 [1.60–2.63]) and antihypertensive medication use (OR 1.98 [1.53–2.58]) compared to class II obesity. Hypertension was associated with lower HRV indices (*p* < 0.001), higher odds of diabetes (OR 4.20 [2.88–6.12]) and dyslipidemia (OR 2.85 [2.17–3.74]), greater use of related medications (OR 3.53 [2.18–5.70)] and 2.96 [1.99–4.40]), respectively), and lower physical activity (OR 0.64 [0.47–0.87]). Physical activity was associated with higher 24 h HRV indices (*p* < 0.001) and lower odds of hypertension (OR 0.64 [0.47–0.87]) and antihypertensive medication use (OR 0.70 [0.50–0.97]). **Conclusions**: Severe obesity and hypertension were associated with reduced 24 h HRV, whereas physical activity was associated with more favorable HRV parameters in adults undergoing evaluation for bariatric surgery, supporting its relevance for cardiovascular health in this population.

## 1. Introduction

The worldwide prevalence of obesity more than doubled between 1990 and 2022. Approximately 16% of adults (~890 million) aged 18 years and older were diagnosed with obesity in 2022 [1]. Severe obesity was extremely rare before the early 1970s, but it has since increased faster than obesity, with no evidence of slowing down [2]. The hazard ratios for cardiovascular disease mortality increase by 42% per 5 kg·m^−1^ units were greater in younger than older people [3]. Hypertension and diabetes mellitus, as well as physical inactivity, are more prevalent, mainly in people with severe obesity [4]. Furthermore, cardiac autonomic dysfunction has recently emerged as a major risk factor for the development of cardiovascular disease, hypertension, and diabetes mellitus [5]. The Framingham Heart Study has provided insight into the prognostic implications of altered heart rate variability (HRV) [6], given that reduced HRV predicted increased risk for subsequent cardiac events in adults free of clinically apparent heart disease.

A meta-analysis demonstrated that sedentary behavior combined with high body mass index (BMI), hypertension, and diabetes mellitus remarkably increased mortality risk [7]. According to the physical activity guidelines of the U.S. Department of Health and Human Services, adults should engage in physical activity for a minimum of 150 min of moderate intensity or 75 min of vigorous intensity per week (min·wk^−1^) [8]. Among American adults, only 24.2% met these physical activity guidelines [9]. The World Health Organization estimated that 1.4 billion adults failed to meet recommended physical activity levels. The number of insufficiently active adults has been reported to be as high as 27.5% worldwide [10] and 33.3% in Brazil [11]. For the total population, meeting at least the minimum physical activity recommendations would potentially prevent nearly one in every two deaths associated with physical inactivity [12]. Further, studies have shown that physical activity improves health outcomes, including measures of HRV [13,14].

HRV varies throughout the day across different physiological states, including daily activities and sleep [15]. However, most studies have assessed HRV using short-term recordings under controlled laboratory conditions [16,17,18,19,20,21], which may not fully reflect autonomic modulation during real-life situations. In contrast, 24 h ambulatory assessment allows for continuous evaluation across these varying conditions, providing greater ecological validity. Ambulatory assessments have been considered important in the prevention and stratification of cardiovascular risks. The 24 h analysis investigates the effects of obesity, hypertension, and physical activity under real-life conditions. The problem is that most studies have been conducted in older individuals [15,22,23,24,25] or with a small number of participants [23,26,27], with a heterogeneous BMI (people with normal weight, overweight and obesity) [24,28,29]; or broad obesity categories, with limited representation (i.e., less than 1% of the participants) [22] or exclusion of individuals with severe obesity [24,26,27,28,30]. As a result, evidence regarding 24 h heart rate variability and autonomic modulation in individuals with severe obesity under real-life conditions remains limited, particularly regarding the potential influence of hypertension and physical activity. This information could enable adequate management and a consequent reduction in cardiovascular risk associated with obesity, mainly due to the world’s increase in obesity and inactive behavior.

This retrospective cross-sectional study based on medical records aimed to investigate the associations of severe obesity, hypertension diagnosis, and physical activity level with 24 h HRV indices in the time and frequency domains in adults undergoing evaluation for bariatric surgery. We hypothesized that severe obesity and hypertension would be associated with impaired ambulatory cardiac autonomic modulation, while physical activity would be associated with improved autonomic function.

## 2. Materials and Methods

### 2.1. Participants

Medical records from 1231 patients referred for preoperative cardiological evaluation for bariatric surgery were analyzed. This setting provided access to a large and clinically well-characterized population of individuals with severe obesity undergoing standardized evaluation. These evaluations were conducted at a specialized clinic located in Cuiabá, Mato Grosso, in the Midwest region of Brazil, and took place between January 2010 and December 2018. The inclusion criteria were the following: ages between 18 and 65 years and both sexes. Exclusion criteria were use of beta-blockers, antiarrhythmics, bronchodilators or other medications that could interfere with heart rate; coronary artery disease, classes III and IV of decompensated congestive heart failure according New York Heart Association; dialysis renal failure; hypothyroidism or hyperthyroidism; chronic obstructive pulmonary disease; asthma or other serious lung diseases; patients with moderate to severe valvular heart disease, dilated cardiomyopathy, idiopathic hypertrophic cardiomyopathy and/or any disease with obstruction of the left ventricular outflow tract; high incidence supraventricular and/or ventricular arrhythmias on 24 h Holter monitoring; sinus node disease; atrioventricular block; patients with acute or subacute vascular brain disease in the last 6 months; sufferers of any disabling chronic illness; Holter patients who did not meet the requirements for an examination of technically adequate quality according to the Recommendations of the Brazilian Society of Cardiac Arrhythmias for Holter Services (minimum of 18 h of recording including wakefulness and sleep; minimum of 3 channels; maximum of 5% artifacts), or exams carried out in other services. The protocol study was conducted in accordance with the standards set by the Declaration of Helsinki and the ethical guidelines of the Ethics Committee in Human Research of the Federal University of Mato Grosso.

The following data were obtained: demographic (age, sex), anthropometrics (body mass, height, BMI), blood pressure (BP), personal history of cardiovascular risk factors (hypertension, diabetes mellitus, dyslipidemia, smoking), use of medications, and information regarding the practice of physical activity. Clinical data and physical activity level were obtained from medical records. All patients were evaluated by the same cardiologist, who is also a co-author of this study, ensuring that diagnostic criteria were known and consistently applied. These criteria are described for transparency and were not defined by the research protocol. Holter recordings were not extracted as structured data from medical records and were analyzed a posteriori by the same cardiologist.

### 2.2. Body Mass Index (BMI)

The patients were classified according to BMI: class II obesity (≥35 e ≤ 39.9 kg·m^−2^) and class III obesity or severe obesity (≥40 kg·m^−2^).

### 2.3. Hypertension Diagnosis

Hypertension was defined based on a previous diagnosis and/or use of antihypertensive medication. Criteria for defining hypertension in the absence of antihypertensive medication, according to International Society of Hypertension Guidelines, were characterized by a persistent elevation in BP, that is systolic BP (SBP) ≥ 140 and/or diastolic BP (DBP) ≥ 90 mmHg, on at least two different occasions [31].

### 2.4. Diabetes Mellitus Diagnosis

Diabetes mellitus was defined based on a previous diagnosis and/or use of hypoglycemic medication. Diabetes Mellitus diagnosis was defined by the criteria of the American Diabetes Association: fasting plasma glucose ≥ 126 mg·dL^−1^, or when 2 h postprandial glucose was ≥200 mg·dL^−1^, or glycated hemoglobin (A1c) ≥ 6.5% [32].

### 2.5. Dyslipidemia Diagnosis

Diagnosis was based on previous history of dyslipidemia and/or use of lipid-lowering medication. Dyslipidemia diagnosis was defined by the criteria of ESC/EAS Guidelines for the Management of Dyslipidemias, when triglycerides ≥ 150 mg·dL^−1^, or total cholesterol ≥ 190 mg·dL^−1^, or low-density lipoprotein ≥ 70 mg·dL^−1^, or high-density lipoprotein ≤ 40 mg·dL^−1^ [33].

### 2.6. Physical Activity Level

Physical activity level was assessed by self-report during the cardiologic visit, based on physical activity guidelines for adults aged 18–64 years [8]. Participants were asked about the frequency, duration, and intensity of their habitual physical activity. Participants were classified as physically active if they reported a minimum 150 min·wk^−1^ of moderate intensity or at least 75 min·wk^−1^ of vigorous intensity activity [8]. Those who did not meet these criteria were classified as physically inactive (non-active group).

### 2.7. 24 h Cardiac Autonomic Modulation

Ambulatory HRV was defined as the continuous recording and analysis of RR intervals (RRi) over a 24 h under free-living conditions using a Holter monitor. A Holter Monitor DMS 300-12T (DMS, São Paulo, Brazil) was placed by fixing electrodes using four precordial derivations to specific anatomical points on the trunk, following the manufacturer’s recommendations. All procedures were performed by the same cardiologist. The Holter monitor had been previously programmed to measure the RRi over a period of 24 h. Heart rate and HRV indices were analyzed using the software package Cardio Scan Premier 11 (DMS, Brazil). All HRV parameters were calculated based on the entire 24 h recording, and the reported values represent global averages. Artifact correction was performed using the software’s automatic filtering procedures, and recordings with inadequate signal quality were excluded from the analysis. The HRV indices evaluated were (1) linear time-domain—the root mean square of successive RR differences (RMSSD), the standard deviation of normal-to-normal RRi (SDNN), standard deviation of the averages of NN intervals in all 5 min segments of the entire recording (SDANN), mean of the standard deviations of all NN intervals for all 5 min segments of the entire recording (SDNNi) and the relative number of successive RRi pairs that differed more than 50 ms (pNN50); (2) frequency domain measured by the Fast Fourier Transform method, using spectral analysis with the area under low-frequency spectral components, LF (with a 0.04 to 0.15 Hz variation); high-frequency spectral components, HF (with a 0.15 to 0.4 Hz variation); LF/HF ratio (ratio between low- and high-frequency components). The power of each spectral component was normalized by dividing the power of each spectrum band by the total power minus the value of the very-low-frequency band and multiplying the result by 100. RMSSD and pNN50 were used as markers of parasympathetic modulation. SDNN reflects overall HRV, representing the combined influence of sympathetic and parasympathetic activity, whereas the SDANN provides an estimate of long-term components of HRV. SDNNi reflects short to intermediate-term HRV, representing autonomic modulation across successive short segments over the 24 h. HF (ms2 and nu) was considered an index of parasympathetic modulation, while the LF/HF ratio was calculated as an indicator of the sympathovagal balance [34], although its physiological interpretation should be made with caution.

### 2.8. Statistical Analysis

Data normality was tested using the Kolmogorov–Smirnov test. Since most variables did not follow a normal distribution, non-parametric tests were applied. Data are presented as median (interquartile range) and minimum–maximum values. Differences in obesity indicators, BP, and cardiac autonomic modulation according to obesity class, hypertension diagnosis, or physical activity level were compared using the Mann–Whitney U test. Categorical variables were analyzed with the Chi-square test. Additionally, the odds ratios of each group being diagnosed with severe obesity, hypertension, diabetes mellitus, dyslipidemia, and medication use were calculated. The Kruskal–Wallis test, followed by Dunn’s post hoc test, was used to measure the interaction of each pair of conditions (obesity class vs. physical activity level; hypertension diagnosis vs. physical activity level; and obesity class vs. hypertension diagnosis) for SDNN values. The BMI was correlated with the SDNN index using Spearman’s Rank correlation. SDNN was selected for correlation analyses as it represents overall HRV over the 24 h period and is considered a robust global index in long-term recordings [34]. The significance level adopted was 5% (*p* ≤ 0.05).

## 3. Results

**Figure 1:**
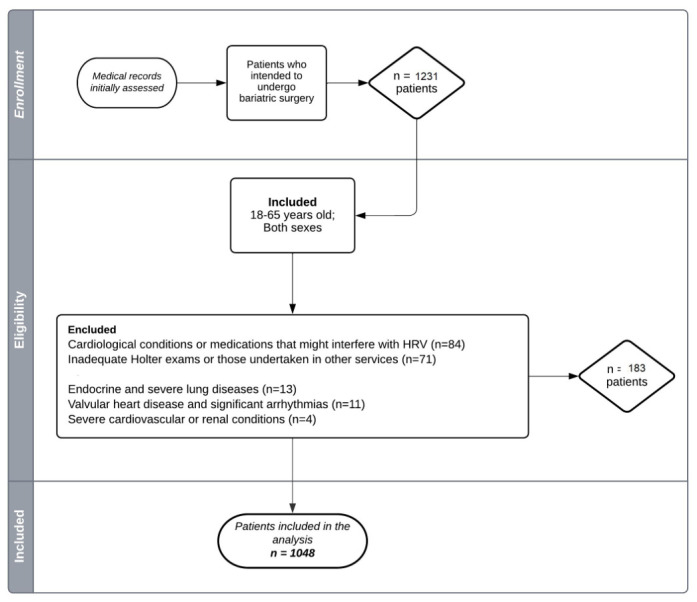
Flow diagram of included and excluded participants.

From the initial sample, 183 were excluded. The most frequent reason for exclusion was cardiological conditions or medications that might interfere with HRV (45.9%), followed by inadequate Holter exams or those undertaken in other services (38.8%). Endocrine and severe lung diseases for 7.1%, valvular heart disease and significant arrhythmias for 6.0%, and severe cardiovascular or renal conditions for 2.2% (Figure 1). Considering all (*n* = 1048) participants, 71.4% were female, and 50.3% had severe obesity. Additionally, 41.7, 14.5, and 29.3% had diagnoses of hypertension, diabetes mellitus, and dyslipidemia, respectively, while 33.0, 8.2, and 11.4% used medications for these conditions. In addition, 19.5% were smokers, and 21.8% were physically active. Most patients (71.3%) were young adults (<40 years old).

Higher values of body mass, BMI and BP (*p* < 0.01) were observed in the group with severe obesity compared to the group with class II obesity. Additionally, 24 h HRV indices, in both time and frequency domain, were lower, and 24 h heart rate was higher (*p* < 0.01) in the group with severe obesity (Table 1). It is noted that patients with severe obesity had approximately twofold higher odds (*p* < 0.01) of hypertension diagnosis (OR 2.04 [1.60–2.63]) and use of antihypertensive medication (OR 1.98 [1.53–2.58]). No differences between the groups (*p* > 0.05) were observed concerning age, diabetes mellitus and dyslipidemia diagnosis, or medication use for these diseases, or smoking status (Table 2). Also, when participants were stratified according to hypertension diagnosis, higher body mass, BMI, and BP values (*p* < 0.01) were observed in the hypertensive group compared with the normotensive group. Moreover, 24 h HRV indices, in both the time and frequency domain were lower, whereas 24 h heart rate was higher (*p* < 0.01) in the hypertensive group (Table 1). Hypertensive patients also had approximately fourfold and threefold higher odds (*p* < 0.01) of diabetes mellitus (OR 4.20 (2.88–6.12)) and dyslipidemia (OR 2.85 [2.17–3.74]), respectively, as well as greater use of medications for these diseases (OR 3.53 [2.18–5.70] and OR 2.96 [1.99–4.40]). Conversely, hypertensive patients had lower odds of being physically active (OR 0.64 [0.47–0.87], *p* < 0.01).

Notably, when we divided the patients according to physical activity level, SBP (*p* < 0.01) was lower, HRV indices (mainly time domain) were higher and the 24 h heart rate was lower (*p* < 0.05) in the physically active group compared to the non-active group (Table 1). The physically active patients had a ~36% lower chance of being diagnosed with hypertension (OR 0.64 (0.47–0.87; *p* < 0.01) and a ~30% lower potential for use of antihypertensive medication (OR 0.70 (0.50–0.97; *p* = 0.02). No differences between the groups (*p* > 0.05) were observed concerning age, diabetes mellitus, and dyslipidemia diagnosis; taking medicines for these diseases; or smoking status (Table 2).

**Figure 2:**
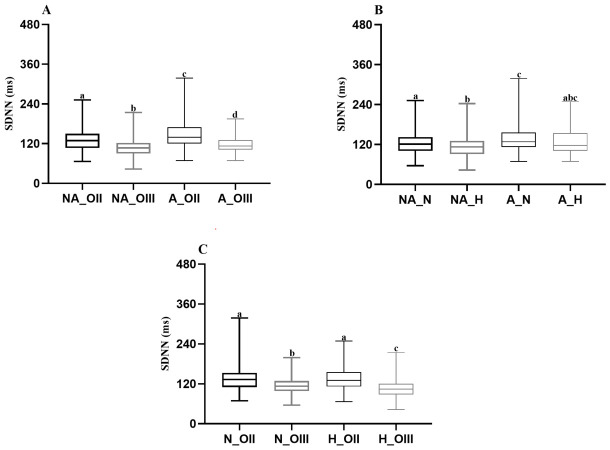
SDNN according to obesity class, hypertension diagnosis, and physical activity level. (**A**) SDNN index between different obesity class and physical activity level: non-active with class II obesity (NA_OII; n = 406), non-active with class III obesity (NA_OIII; n = 412), active with class II obesity (A_OII; n = 118), and active with class III obesity (A_OIII; n = 112). (**B**) SDNN index according to hypertension diagnosis and physical activity level: non-active normotensive (NA_N; n = 459), non-active hypertensive (NA_H; n = 359), active normotensive (A_N; n = 153), and active hypertensive (A_H; n = 77). (**C**) SDNN index according to obesity class and hypertension diagnosis: normotensive with class II obesity (N_OII; n = 351), normotensive with class III obesity (N_OIII; n = 261), hypertensive with class II obesity (H_OII; n = 173), hypertensive with class III obesity (H_OIII; n = 263). Data are presented as median, interquartile range, and minimum–maximum values and were analyzed by the Kruskal–Wallis test followed by Dunn’s post hoc test. Different letters indicate statistically significant differences between groups (*p* ≤ 0.05).

When analyzing the interaction between obesity class and physical activity level, a significant interaction effect was observed (*p* < 0.05). Participants with severe obesity who were non-active (107 ms) exhibited lower SDNN values compared to non-active individuals with class II obesity (129 ms). In contrast, physically active participants showed higher SDNN values in both severe obesity (113 ms) and class II obesity (139.2 ms), with the highest values observed in the physically active class II obesity group (Figure 2A).

Additionally, analysis of the interaction between hypertension diagnosis and physical activity level revealed a significant effect (*p* < 0.05). Hypertensive non-active participants (113 ms) exhibited lower SDNN values compared to non-active normotensive individuals (121 ms). However, physically active hypertensive participants (117 ms) showed SDNN values comparable to those of the other groups (Figure 2B).

When analyzing the interaction between obesity class and hypertension diagnosis, a significant interaction effect was observed. Normotensive participants with severe obesity exhibited lower SDNN values compared to those with normotensive participants with class II obesity and (113 vs. 133 ms, *p* < 0.001). Similarly, hypertensive participants with severe obesity showed lower SDNN values than hypertensive participants with class II obesity (104 vs. 130 ms, *p* < 0.001). In addition, within the severe obesity group, hypertensive participants exhibited lower SDNN values compared to normotensive participants (104 vs. 113 ms, *p* < 0.001). However, SDNN values were similar between hypertensive and normotensive participants with class II obesity (130 vs. 133 ms) (Figure 2C).

**Figure 3:**
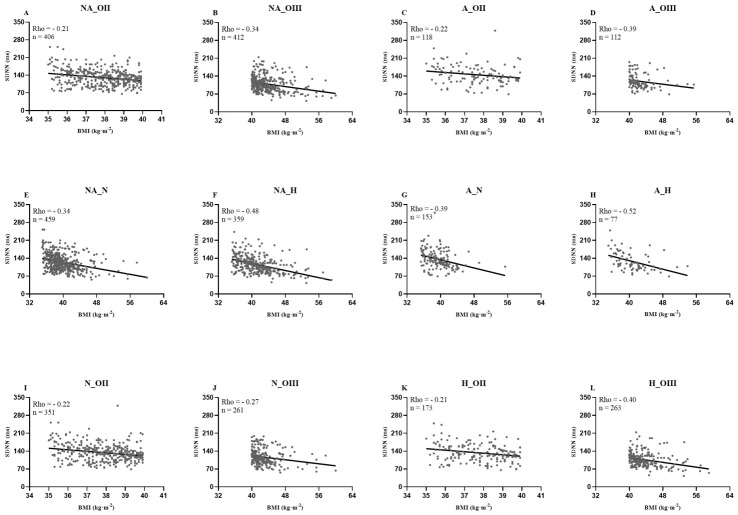
Correlation of SDNN with BMI according to obesity class, hypertension diagnosis and physical activity level. The SDNN was correlated with BMI in the following groups: (**A**) non-active with class II obesity (NA_OII), (**B**) non-active with class III obesity (NA_OIII), (**C**) active with class II obesity (A_OII), (**D**) active with class III obesity (A_OIII), (**E**) non-active normotensive (NA_N), (**F**) non-active hypertensive (NA_H), (**G**) active normotensive (A_N), (**H**) active hypertensive (A_H), (**I**) normotensive with class II obesity (N_OII), (**J**) normotensive with class III obesity (N_OIII), (**K**) hypertensive with class II obesity (H_OII), and (**L**) hypertensive with class III obesity (H_OIII). The correlation was assessed using Spearman’s Rank correlation test.

BMI was significantly correlated with SDNN (*p* < 0.01) in the participants with class II obesity (Rho = −0.21 and −0.22) and severe obesity (Rho = −0.34 and −0.39) in the non-active and active groups, respectively (Figure 3A–D). In addition, BMI was significantly correlated with SDNN (*p* < 0.01) in normotensive (Rho = −0.34 and −0.39) and hypertensive participants (Rho = −0.48 and −0.52) in the non-active and active groups, respectively (Figure 3E–H). Correlation coefficients were stronger in hypertensive participants, but similar across physical activity levels. BMI was significantly correlated with SDNN (*p* < 0.01) in class II obesity (Rho = −0.22 and −0.21) and severe obesity (Rho = −0.27 and −0.40) in normotensive and hypertensive participants, respectively (Figure 3I–L).

## 4. Discussion

The present study suggests that severe obesity and hypertension are associated with impaired ambulatory cardiac autonomic modulation in individuals undergoing evaluation for bariatric surgery. Conversely, physical activity is associated with better cardiac autonomic modulation, even in the presence of severe obesity and hypertension reinforcing the potential protective role of physical activity in this high cardiovascular risk population.

To our knowledge, this is the first large retrospective cross-sectional study to examine the association of severe obesity, hypertension, and physical activity on 24 h time and frequency domain indices of HRV among adults with obesity. Additionally, detailed information regarding multiple cardiovascular risk factors, including diabetes mellitus, dyslipidemia, medication use, and smoking, was available, allowing for the assessment of their associations with the outcomes using odds ratio analyses. In some important studies with large sizes, the population studied consisted predominantly of middle-aged individuals (~45 and 50 years) [16,23,24,30], or older (~60 and 70 years) [15,22], with a heterogeneous BMI (people with normal weight, overweight, and obesity). Furthermore, individuals with severe obesity were either markedly underrepresented (<1% of participants) [22], or excluded [24,26,27,28,30], and several studies were limited to short-term HRV assessment [16,18,19,20,21] rather than 24 h monitoring. The results of this study emphasized the importance of 24 h continuous monitoring of autonomic variables, especially in patients with obesity. An understanding of obesity, hypertension, and physical activity on ambulatory cardiac autonomic modulation is essential for successful prevention and treatment strategies.

According to the Framingham Heart Study, a one-standard-deviation decrease in the SDNN was associated with a hazard ratio of 1.47 for new cardiac events in adults free of clinically apparent heart disease [6]. Both a reduced vagal function and the imbalance in the sympathovagal function were associated with the risk of developing hypertension [17]. In the same way, autonomic function was affected early in patients with diabetes, occurring before the advent of traditional risk factors and markers of inflammation [35]. In addition, a meta-analysis found that higher BMI and a greater percentage of hypertension and diabetes further increased all-cause mortality risk in the most sedentary populations, while at the same time, higher physical activity levels decreased the rate of mortality [8]. Another meta-analysis, including cohort studies, found that the risk of hypertension was reduced by 6% for those who met the recommended guidelines for minimum physical activity levels of 150 min·wk^−1^ [35]. In another study, with over 15 years of follow-up [36], those who were more versus less physically active experienced a 17% reduced risk for incident hypertension, after adjustment for race, sex, age, education, and family history of high BP in young adults.

Apart from obesity as a potential contributor to unfavorable autonomic function, other studies have shown that increased physical activity can improve cardiac autonomic modulation. Regardless of weight, the improvement in SDNN (~18–19%) was greatest among those who exercised regularly. The difference in SDNN within each BMI category between regularly exercising and sedentary subjects was highest in individuals with obesity [22]. Likewise, lifetime physical activity explained 40% of the variance in SDNN in adults with obesity [37]. Each one-category increase in the physical activity index increased the SDNN by 15.4%. High VO2max explained 45 and 25% of the variance in HF and LF/HF ratio, respectively [37].

The mechanisms through which obesity causes HRV impairment and elevated BP have still not been elucidated. Activation of the sympathetic nervous system has been considered to have a key function in the pathogenesis of obesity-related hypertension [38]. A chronically elevated sympathetic nervous system outflow could, in turn, impair β-adrenergic signaling, reduce metabolism stimulation, and evolve into a cycle that contributes to obesity and insulin resistance [39]. Inflammation (i.e., C-reactive protein, interleukin-6, and tumor necrosis factor-α) and insulin resistance may alter the vascular function and consequently impair BP and HRV. In addition, plasma renin activity, angiotensinogen, angiotensin II, and aldosterone values are increased during obesity [38]. On the other hand, another study indicated that within the same BMI category, the group that met the physical activity recommendation had lower C-reactive protein [40]. In this way, Jae et al. showed that the association between higher cardiorespiratory fitness and lower C-reactive protein levels is dependent on autonomic function [41]. Vagal activity seems to have an anti-inflammatory effect via a cholinergic pathway, which results in a down-regulation of pro-inflammatory cytokines [42]. In this sense, reduced autonomic modulation, particularly decreased parasympathetic modulation, has been consistently reported in individuals with obesity [23]. In addition, reduced HRV may reflect a global attenuation of autonomic responsiveness rather than a simple shift toward sympathetic predominance [34].

The findings of the Pope et al. [18] study suggested that vigorous-intensity and light-intensity physical activity have independent positive associations with SDNN and/or RMSSD index following robust adjustment for relevant intermediates and that fasting insulin was a consistent physiological mediator for these associations. In a previous study by our research group [14], young adults who met at least the minimum physical activity recommendations (150 min·wk^−1^) showed a higher cardiac autonomic modulation, even without BMI differences. These studies [14,18] used HRV data obtained from short analysis recordings and did not encapsulate the effects of circadian rhythm and daily activity, in contrast to longer recordings such as those used in our study.

Our findings indicate that obesity severity is the main determinant of cardiac autonomic dysfunction in individuals with obesity undergoing evaluation for bariatric surgery. Initial analyses across HRV indices showed consistently worse autonomic profiles with increasing obesity, hypertension, and physical inactivity. Focusing on SDNN, interaction analyses revealed that the combination of severe obesity and hypertension was associated with the lowest values, whereas physical activity was linked to more favorable profiles, although it did not fully offset the effects of severe obesity. Correlation analyses further supported these findings, demonstrating an inverse dose–response relationship between BMI and HRV, which was stronger in individuals with hypertension and similar across physical activity levels. Overall, these findings suggest that obesity severity may be the primary driver of autonomic dysfunction, while hypertension may act as an aggravating factor and physical activity as a protective moderator.

Our findings have relevant clinical and public health implications. Both a lower obesity class and meeting physical activity recommendations were associated with more favorable 24 h HRV profiles. However, given the difficulty in achieving and maintaining weight loss, physical activity may represent a more feasible strategy to improve cardiac autonomic modulation in individuals with severe obesity. Accordingly, physically active individuals consistently exhibited higher HRV across groups. While current guidelines recommend at least 150 min·wk^−1^ of moderate-intensity physical activity, further studies are needed to compare the relative contributions of physical activity and weight loss to HRV improvement.

Some limitations of our study should be acknowledged: (1) The retrospective cross-sectional design precluded causal inferences. (2) The physical activity level was assessed using self-reported measures rather than objective methods such as accelerometry. (3) Visceral adiposity measures, such as waist circumference, which may be more strongly associated with cardiovascular risk and autonomic dysfunction, was not assessed. (4) We did not have the data concerning antihypertensive therapy. (5) Respiratory rate was not monitored during the 24 h recordings, since respiratory patterns cannot be standardized under ambulatory free-living conditions, which may affect HRV indices, particularly frequency-domain measures. (6) The study population consisted of patients undergoing preoperative cardiological evaluation for bariatric surgery, which may limit the generalizability of the findings. (7) Serum insulin and inflammatory markers were not assessed. (8) The data were originally collected from clinical records. Therefore, several factors known to influence HRV were not controlled, including recent vigorous physical activity, alcohol intake, caffeine consumption, sleep quality, mental stress, and psychiatric or neurological conditions during the monitoring period.

## 5. Conclusions

In summary, severe obesity and hypertension were associated with reduced 24 h HRV, whereas physical activity was associated with more favorable HRV parameters in adults undergoing evaluation for bariatric surgery. Our findings support the relevance of physical activity for cardiovascular health in this population.

## Figures and Tables

**Table 1:** Obesity indicators, clinic blood pressure, and 24 h cardiac autonomic modulation variables according to obesity class, hypertension diagnosis, and physical activity level.

	All *n* = 1048	Class II Obesity *n* = 524	Severe Obesity *n* = 524	Normotensive *n* = 612	Hypertensive *n* = 436	Non-Active *n* = 818	Active *n =* 230
Age (years)	34 (28, 41)	34 (29, 40)	34 (28, 42)	32 (27, 37)	37 (30, 46) **^#^**	34 (28, 41)	34 (29, 41)
Body mass (kg)	109 (100, 120)	102 (97, 109)	117 (108, 129) *	106 (99, 115)	114 (103, 128) **^#^**	109 (100, 121)	108 (99, 119)
BMI (kg·m^−2^)	40 (38, 42)	38 (37, 39)	42 (41, 44) *	39 (38, 41)	41 (38, 43) **^#^**	40 (38, 42)	40 (38, 42)
		Rest					
Systolic BP (mmHg)	127 (121, 135)	126 (120, 134)	129 (122, 137) *	123 (117, 128)	135 (128, 143) **^#^**	128 (121, 136)	125 (120, 132) **^$^**
Diastolic BP (mmHg)	84 (80, 88)	84 (80, 88)	85 (81, 89) *	82 (77, 87)	87 (82, 92) **^#^**	84 (80, 88)	84 (80, 88)
		Holter 24 h					
RMSSD (ms)	31 (24, 39)	33 (25, 41)	28 (22, 36) *	32 (24, 41)	29 (22, 36) **^#^**	30 (23, 38)	33 (24, 43) **^$^**
SDNN (ms)	119 (99, 142)	132 (110, 153)	109 (92, 123) *	123 (103, 147)	114 (93, 136) **^#^**	117 (97, 139)	125 (109, 154) **^$^**
SDANN (ms)	108 (88, 130)	119 (99, 143)	99 (82, 113) *	112 (93, 132)	103 (84, 124) **^#^**	106 (86, 127)	113 (95, 141) **^$^**
SDNNi (ms)	49 (40, 61)	53 (43, 66)	46 (38, 56) *	51 (43, 65)	46 (38, 59) **^#^**	48 (39, 60)	54 (44, 69) **^$^**
pNN50 (%)	8 (3, 16)	10 (4, 18)	6 (3, 13)*	9 (4, 17)	7 (3, 13) **^#^**	8 (3, 15)	10 (3, 20) **^$^**
HF (ms^2^)	204 (122, 370)	261 (161, 432)	158 (98, 303) *	236 (142, 421)	179 (110, 317) **^#^**	197 (118, 344)	244 (144, 461) **^$^**
HF (n. u.)	0.29 (0.22, 0.36)	0.30 (0.24, 3.7)	0.28 (0.21, 0.35) *	0.30 (0.23, 0.37)	0.28 (0.22, 0.35) **^#^**	0.29 (0.22, 0.37)	0.29 (0.23, 0.36)
LF/HF	2.4 (1.8, 3.5)	2.3 (1.7, 3.2)	2.6 (1.8, 3.9) *	2.3 (1.7, 3.3)	2.6 (1.8, 3.6) **^#^**	2.4 (1.7, 3.5)	2.5 (1.8, 3.3)
Heart rate (bpm)	81 (75, 87)	79 (73, 86)	83 (77, 88) *	80 (74, 87)	82 (76, 88) **^#^**	82 (76, 88)	75 (70, 83) **^$^**

The data are presented as median (interquartile range) and were analyzed using the Mann–Whitney U test; n represents the number of participants in each group. (*) *p* < 0.01 difference from class II obesity, (**^#^**) *p* < 0.01 difference from normotensive, (**^$^**) *p* < 0.01 difference from non-active. BMI: Body mass index; BP: blood pressure.

**Table 2:** Risk factors for cardiovascular diseases according to obesity class, hypertension diagnosis, and physical activity level.

	All *n* = 1048	Class II Obesity *n* = 524	Severe Obesity *n* = 524	Normotensive *n* = 612	Hypertensive *n* = 436	Non-Active *n* = 818	Active *n* = 230
Sex (F/M)	71.5% (749/299)	77.1% (404/120)	65.8% (345/179) *	83.3% (510/102)	54.8% (239/197) **^#^**	72.4% (592/226)	68.3% (157/73)
Age range (years)							
18–29	30.5% (320)	29.6% (156)	31.3% (164) *	36.9% (227)	21.6% (94) **^#^**	30.9% (253)	29.1% (67)
30–39	40.7% (427)	43.65% (228)	38.0% (199)	43.5% (266)	36.9% (161)	40.3% (330)	42.2% (97)
40–49	19.6% (205)	18.9% (99)	20.2% (106)	16.0% (98)	24.5% (107)	19.8% (162)	18.7% (43)
50–59	7.6% (80)	47.6% (40)	7.6% (40)	3.3% (20)	13.8% (60)	7.6% (62)	7.8% (18)
60–65	1.5% (16)	0.2% (1)	2.9% (15)	0.3% (2)	3.2% (14)	1.3% (11)	2.2% (5)
Severe obesity	50.0% (524)	–	–	42.6% (261)	60.3% (263) **^#^**	50.4% (412)	49.6%(112)
Hypertension	41.7% (436)	33.0% (173)	50.2% (263) *	*–*	*–*	43.9% (359)	33.5% (77) **^$^**
Hypertension medication	32.8% (344)	25.4% (133)	40.3% (214) *	0.3% (2)	78.4% (342) **^#^**	34.5% (282)	27.0% (62) **^$^**
DM	14.4% (151)	13.9% (73)	14.9% (78)	7.2% (44)	24.5% (107) **^#^**	14.1% (115)	15.7% (36)
DM medication	8.1% (85)	9% (47)	7.3% (38)	4.2% (26)	13.5% (59) **^#^**	7.9% (65)	8.7% (20)
Dyslipidemia	29.3% (307)	30.3% (159)	28.2% (148)	20.3% (124)	42.0% (183) **^#^**	30.0% (245)	27.0% (62)
Dyslipidemia medicine	11.4% (120)	13.2% (69)	9.7% (51) *	6.9% (42)	17.9% (78) **^#^**	11.9% (97)	10.0% (23)
Smoking status	19.6% (205)	20.4% (107)	18.7% (98)	17.2% (105)	22.9% (100) **^#^**	19.7% (161)	19.1% (44)
Physically active	21.9% (230)	22.5% (118)	21.4% (112)	25.0% (153)	17.7% (77) **^#^**	–	–

The data were presented as percentage (number of participants in each group) and were compared using the Chi-square test. (*) *p* ≤ 0.05 difference from class II obesity, (**^#^**) *p* < 0.01 difference from normotensive, (**^$^**) *p* < 0.05 difference from non-active. DM: diabetes mellitus.

## Data Availability

The data that support the findings of this study are available from the corresponding author upon reasonable request.

## Abbreviations

The following abbreviations are used in this manuscript:

BMI	body mass index
BP	blood pressure
DBP	diastolic blood pressure
DM	diabetes mellitus
HR	heart rate
HF	high-frequency component
HRV	heart rate variability
LF	low-frequency component
OR	odds ratio
pNN50	relative number of successive RRi pairs that differed more than 50 ms
RMSSD	root mean square of successive RR differences
SBP	systolic blood pressure
SDANN	standard deviation of the averages of NN intervals in all 5 min segments of the entire recording
SDNN	standard deviation of normal-to-normal RRi
SDNNi	mean of the standard deviations of all NN intervals for all 5 min segments of the entire recording
RRi	RR intervals
